# Human-Technology Interaction Factors Associated With the Use of Electronic Personal Health Records Among Younger and Older Adults: Secondary Data Analysis

**DOI:** 10.2196/27966

**Published:** 2021-10-26

**Authors:** Yan Luo, Krystal Dozier, Carin Ikenberg

**Affiliations:** 1 School of Social Work The University of Alabama Tuscaloosa, AL United States

**Keywords:** electronic personal health records, human-technology interaction factors, clinical notes, smartphone app, ease of understanding

## Abstract

**Background:**

An electronic personal health record (ePHR), also known as a personal health record (PHR), has been broadly defined as an electronic application through which individuals can access, manage, and share their health information in a secure and confidential environment. Although ePHRs can benefit individuals as well as caregivers and health care providers, the use of ePHRs among individuals continues to remain low.

**Objective:**

The current study aims to examine the relationship between human-technology interaction factors and ePHR use among adults and then to compare the different effects of human-technology interaction factors on ePHR use between younger adults (18-54 years old) and older adults (55 years of age and over).

**Methods:**

We analyzed data from the Health Information National Trends Survey (HINTS 5 cycle 3) collected from US adults aged 18 years old and over in 2019. Descriptive analysis was conducted for all variables and each item of ePHR use. Bivariate tests (Pearson correlation coefficient for categorical variable and *F* test for continuous variables) were conducted over 2 age groups. Finally, after adjustments were made for sociodemographics and health care resources, a weighted multiple linear regression was conducted to examine the relationship between human-technology interaction factors and ePHR use.

**Results:**

The final sample size of 1363 (average age 51.19) was divided into 2 age groups: 18 to 54 years old and 55 years old and older. The average level of ePHR use was low (mean 2.76, range 0-8). There was no significant difference in average ePHR use between the 2 age groups. Including clinical notes was positively related to ePHR use in both groups: 18 to 54 years old (*β=.28, P*=.005)*,* 55 years old and older (*β*=.15*, P*=.006). Although accessing ePHRs using a smartphone app was only associated with ePHR use among younger adults (*β*=.29*; P*<.001), ease of understanding health information in ePHRs was positively linked to ePHR use only among older adults (*β=*.13; *P*=.003).

**Conclusions:**

This study found that including clinical notes was positively related to ePHR use in both age groups, which suggested that including clinical notes as a part of ePHRs might improve the effective use of ePHRs among patients. Moreover, accessing ePHRs using a smartphone app was associated with higher ePHR use among younger adults while ease of understanding health information in ePHRs was linked to higher ePHR use among older adults. The design of ePHRs should provide the option of being accessible through mobile devices to promote greater ePHR use among young people. For older adults, providers could add additional notes to explain the health information recorded in the ePHRs.

## Introduction

### Electronic Personal Health Record and Its Functions

An electronic personal health record (ePHR), also known as a personal health record (PHR), was broadly defined by the Markle Foundation (2004) to be an electronic application through which individuals can access, manage, and share their health information in a secure and confidential environment [1]. Unlike the electronic health record that is managed by health care providers, an ePHR is managed by individuals [2]. Pagliari and colleagues [3] summarized 7 potential functions of ePHRs: (1) access to health care providers’ electronic clinical records (eg, history, drugs, test results); (2) personal health organizer or diary (eg, clinics, doctors, tests, dates, nonprescribed treatments, scanned documents); (3) self-management support (eg, care plans, graphing of symptoms, passive biofeedback, tailored instructive or motivational feedback, decision aids, or reminders); (4) secure patient-provider communication for scheduling appointments, reordering prescriptions, or seeking advice (eg, patient-doctor email); (5) links to static or informative information about illnesses, treatments, or self-care; (6) links to sources of support; and (7) collective data on symptom or health behavior data by self-report or objective monitoring through electronic devices.

### Benefits of Using ePHRs

Using ePHRs allows individuals to access and coordinate their health information and to share appropriate parts to those who need it [1]. The use of ePHRs can benefit individuals as well as caregivers and health care providers [2,3]. For individuals, ePHRs provide them with credible health information, data, and guidance on potential ways to self-manage diseases and improve health, which facilitates collaborative disease tracking and improved communication between individuals (or their caregivers) and health care providers [2,3]. Moreover, ePHRs provide health care providers with more data on individuals, which allows the provider to make informed decisions, as well as improve the efficiency of care by empowering individuals’ active involvement in health care and enabling PHR-mediated electronic communication [2,3]. For payers and purchasers of health care, the use of ePHRs has the potential to lower costs on chronic disease management, medications, and wellness programs [2]. Several studies have been conducted to evaluate some of the benefits of using ePHRs [4]. For example, a clinical trial testing the effects of ePHRs on advance care planning delivery in primary care settings revealed that using ePHRs improved advanced care planning documentation and quality, especially among patients between 50 and 60 years of age [5]. Another study evaluated the impact of using a decision module through ePHRs to inform cancer screening and demonstrated that participants’ decision on cancer screening can be proactively facilitated through an ePHR decision module [6]. Aside from primary care and preventive care settings, ePHRs also play a positive role in mental health care settings. In a study that compared scores of Patient Health Questionnaire 9 between participants who used ePHRs with their collaborative care managers and those who did not use ePHRs, Pecina and colleagues [7] suggested that ePHR users had a higher number of contacts with care managers and showed higher depression remission.

### ePHR Use and Age Disparity

Although ePHR use has been promoted and health care providers have offered most of their patients access to ePHRs [8], the use of ePHRs among individuals continues to remain low. Using data from the Health Information National Trends Surveys (HINTS) in 2008, 2011, and 2013, a study predicted that the ePHR adoption rate would exceed 75% by 2020 [9]. Although the most recent data of the ePHR use rate in 2020 are not available, a study analyzing data from the HINTS data set in 2018 reported that the use of ePHRs in the United States was only 31.4% [10]. The relationship between age and ePHR use has been documented in previous studies, which indicate that younger age is related to higher ePHR use and that patients who are younger are more likely to use ePHRs [11,12]. Pagliari and colleagues [3] pointed out that older adults had poor technical skills that might cause access disparities regarding use of ePHRs. When encountering technology, older adults face physical or cognitive challenges [13-15]. Age-related changes in functional abilities, such as sight loss, hearing loss, decreased kinesthetic ability, and decreased psychomotor and cognitive skills pose barriers for older adults to use technology [13,14,16]. Previous studies have documented barriers to adopting ePHRs among older adults, including a lack of confidence in the ability to use technology [17], concerns related to privacy [18], problems with access to computers or devices and the PHR system [15], and low health literacy or computer literacy [15]. Examining the human-technology interaction factors associated with ePHR use among individuals in different age groups might inspire tailored ePHR design and training regarding ePHR use among people from different age groups.

After reviewing 97 studies regarding factors that affect the use of ePHRs among patients, a systematic review identified 3 human-technology interaction factors that affect ePHR use: perceived usefulness (positively), internet access (positively), and privacy and security concerns (negatively) [19]. Previous studies have also suggested that other human-technology interaction factors including perceived ease of use [20,21], difficulty getting onto the system [22], and response costs [23] are associated with the use of ePHRs; however, Abd-Alrazaq and colleagues [19] believe that more evidence is needed to draw a firm conclusion regarding these factors.

**This Study**

Broadly, the use of ePHRs provides benefits not only to individuals and caregivers, but also health care providers. Thus, the US Department of Health and Human Services has made investments and efforts to improve ePHR use [8]. Despite the low rate of ePHR use and the digital divide between different age groups, previous studies have not investigated the different effects of human-technology interaction factors on ePHR use between different age groups. Controlling for sociodemographics and health care resources, the current study aims to examine the relationship between human-technology interaction factors and ePHR use among adults and to then compare the different effects of human-technology interaction factors on ePHR use between younger adults (18-54 years old) and older adults (55 years old and older). Although 65 years is widely used as a cutoff point for older adults, using 55 years as the cutoff point in this study was based on previous literature on technology use among older adults. In the United States, the National Telecommunications and Information Administration (2011) used 55 years as a cutoff point and reported that older Americans aged 55 years and older had the lowest adoption rate of broadband [24]. Moreover, a European project, “ICT 4 the Elderly”, developed to improve older adults’ digital skills, also defined older adults using 55 years as a cutoff point [25]. Other literature that has studied the use of health information technology among older adults has used 55 as a cutoff point as well [10,26,27].

## Methods

### Data Collection

In this study, we used the most recent iteration of the HINTS 5 (cycle 3) [28] collected from US adults aged 18 years old and over in 2019. HINTS is a national representative data set from the National Cancer Institute, and it routinely collects data about the American public’s knowledge of, attitudes toward, and use of cancer- and health-related information. Since 2003, HINTS has been used by researchers to understand health communication through the internet in the information age among American adults. Two-stage sampling strategy and two-sampling strata (high- and low-minority strata) were applied during the data collection phase. Random samples of household addresses were selected in the first stage, and 1 adult within each sampled household was randomly selected in the second stage. All selected households received a total of 4 mailings: an initial mailing with a US $2 incentive, a reminder postcard, and 2 follow-up mailings. Participants were provided with 2 toll-free phone numbers (for English and Spanish calls) if they had questions, concerns, or requests for the Spanish survey. Each returned questionnaire was scanned, verified, cleaned, and edited. The final sample yielded 4448 potential respondents with a response rate of 30.2% (4448/14,730) and 3370 completed questionnaires. Only participants who had accessed their ePHRs at least 1 time in the past 12 months were included in this study. The final sample size of 1363 was divided into 2 age groups: 18 to 54 years old and 55 years old and older.

### Dependent Variable

The dependent variable of interest was ePHR use. Participants who accessed their ePHRs at least once in the past 12 months were asked if in the past 12 months they used their online medical record to do any of the following: request a refill of medications; look up test results; request correction of inaccurate information; securely message health care provider and staff; download health information to a computer or mobile device, such as a cell phone or tablet; add health information, such as health concerns, symptoms, and side effects, to share with a health care provider; and help make decisions about how to treat an illness or condition. Each item was answered with a yes or no response by respondents (0=no, 1=yes). The eighth item of ePHR use pertained to sending health information electronically. Participants were asked if they had electronically sent their medical information to another health care provider, to a family member or another person involved with their care, or to a service or app that could help manage and store their health information. This response was also answered with a yes or no response for each option. Participants who selected yes on one of the options were coded as yes on sending health information electronically, while participants who selected no on all 3 options were coded as no on sending health information electronically (0=no, 1=yes). The total ePHR use score was obtained by summing up all 8 items and was analyzed as a continuous variable (range from 0 to 8).

### Sociodemographics and Health Care Resources

#### Sociodemographics

Sociodemographic variables including gender (0=male, 1=female), urbanity (0=rural, 1=urban), and educational attainment (0=below bachelor’s degree, 1=bachelor’s degree and above) were included.

#### Health Care Resources

Having a regular health care provider (0=no, 1=yes) and frequency of visiting health care providers in the past 12 months (0=0-3 times, 1=4 times and above) were included. Having family or friends to talk to about health was also included and analyzed as a dichotomous variable (0=no, 1=yes).

### Human-Technology Interaction Factors

#### Including Clinical Notes

Respondents were asked the following: “Do any of your online medical records include clinical notes (health provider’s notes that describe a visit)?”, with responses yes, no, and “don’t know.” After responses of no and “don’t know” were combined into 1 category, a dichotomous variable was obtained (0=no/don’t know, 1=yes).

#### Ease of Understanding

To determine ease of understating, a 4-point scale was used for participant responses to the following question: “How easy or difficult was it to understand the health information in your online medical records?” Ease of understanding was analyzed as a continuous variable ranging from 0 to 3 (0=very difficult, 1=somewhat difficult, 2=somewhat easy, 3=very easy).

#### Access via Smartphone App

Respondents were asked the following question: “Did you use a smartphone health app to access your online medical record?”, with responses categorized as yes, no, and “don’t know.” Responses of no and “don’t know” were grouped into 1 category; thus, a dichotomous variable was used for the accessibility of a smartphone app (0=no/don’t know, 1=yes).

### Statistical Analysis

Three researchers in this study devised the statistical analysis plan, and the statistical analyses were conducted by YL. The results and interpretation were reviewed by KD and CI. As the complex sampling procedure was applied in the HINTS data collection, the data analysis in this study was conducted using STATA/SE 5.1 (StataCorp), which allowed for incorporating the jackknife replicate weights to assess variation estimation. Descriptive analysis was conducted for all variables and each item of ePHR use. Bivariate tests (Pearson correlation coefficient for categorical variables and *F* test for continuous variables) were conducted over the 2 age groups. Finally, after sociodemographics and health care resource factors were adjusted for, a weighted multiple linear regression was conducted to examine the relationship between human-technology interaction factors and ePHR use. The final sample weight was used to obtain population estimates, and 50 jackknife replicate weights were used to obtain variation estimates. Listwise deletion of participants was also applied in all analyses.

## Results

### Description of Sociodemographics, Health Care Resources, and Human-Technology Interaction Factors

The average age of all participants was 51.18 years. According to Table 1, more than half of the participants were female (762/1266, 57.12% weighted), and less than half of the participants had a bachelor’s degree or above (790/1334, 39. 91% weighted). The majority of participants were from urban areas (1245/1363, 89.75% weighted). In terms of health care resources, more than three-quarters of the participants had a regular health care provider (1091/1339, 78.35% weighted), and around half of the participants visited health care providers more than 4 times in the past 12 months (690/1346, 49.43%). Most of participants reported that they had friends or family to talk to about health (1167/1332, 85.62% weighted). With regard to human-technology interaction factors of ePHRs, about 40% of participants reported that they accessed their ePHRs using a smartphone app (436/1290, 39.56% weighted), and half of the participants said their ePHRs included clinical notes (650/1278, 50.34% weighted). Participants tended to report that it was easy to understand health information in ePHRs (mean 2.31, range 0-3). Table 1 also shows the significant differences in having a regular provider and accessing ePHRs using a smartphone app between the 2 age groups (*P*<.001). More older adults (55 years old and older) reported having a regular health provider, while more younger adults (18-54 years old) reported accessing ePHRs using a smartphone app.

**Table 1:** Description of sociodemographics, health care resources, and human-technology interaction factors (N=1363).

Characteristic							All			18-54 years old			55 years old and older			*P* value	
**Sociodemographics, n (%)**																	
	**Gender**																
				Male		504 (42.88)			190 (41.4)			309 (45.7)			.27		
				Female		762 (57.12)			342 (58.6)			414 (54.3)					
	**Urbanity**																.99
				Rural		118 (10.25)			46 (10.2)			68 (10.3)					
				Urban		1245 (89.75)			505 (89.8)			706 (89.7)					
	**Education**																.10
				Below Bachelor’s degree		544 (60.09)			182 (58.0)			354 (64.2)					
				Bachelor’s degree and above		790 (39.91)			369 (42.1)			416 (35.8)					
**Health care resources, n (%)**																	
	**Regular health care provider**																<.001
				No		248 (21.65)			150 (27.0)			86 (11.4)					
				Yes		1091 (78.35)			395 (73.0)			676 (88.6)					
	**Frequency of visiting health care provider**																.47
				0-3 times in the past 12 months		656 (50.57)			297 (51.6)			334 (48.5)					
				4 times and above in the past 12 months		690 (49.43)			253 (48.4)			421 (51.5)					
	**Having friends/family to talk to about health**																.41
				No		165 (14.38)			68 (13.5)			91 (16.2)					
				Yes		1167 (85.62)			481 (86.5)			666 (83.8)					
**Human-technology interaction factors, n (%)**																	
		**Accessing ePHRs^a^ using smartphone app**														<.001	
			No/don’t know		854 (60.44)			305 (54.8)			528 (71.8)						
			Yes		436 (39.56)			238 (45.2)			193 (28.2)						
		**ePHRs include clinical notes**														.40	
			No/don’t know		628 (49.66)			265 (51.1)			345 (46.8)						
			Yes		650 (50.34)			275 (48.9)			369 (53.2)						
Ease of understanding ePHRs health information (range 0-3), mean							2.31			2.33			2.28			.42	

^a^ePHRs: electronic personal health records.

### Description of ePHR Use

Participants’ ePHR use is reported in Table 2. The average level of ePHR use was low (mean 2.76, range 0-8). There was no significant difference in average ePHR use between the 2 age groups. Table 2 also shows the rate on each item of ePHR use. Specifically, the majority of participants used ePHRs to look up test results (1081/1277, 84.59% weighted). Around half of the participants used ePHRs to request a refill of medications (596/1276, 46.57% weighted) and securely message health care provider and staff (686/1278, 52.96% weighted). About one-quarter of participants used ePHRs to download health information to a computer or mobile device (292/1276, 25.88% weighted), add health information to share with health care providers (307/1278, 23.58% weighted), and help make a decision about how to treat an illness or condition (324/1274, 24.77% weighted). A small percentage of participants used ePHRs to request correction of inaccurate information (104/1263, 7.58% weighted) and electronically send health information (108/1268, 9.62% weighted). Significant differences between the 2 age groups were found related to using ePHRs to “download health information to a computer or mobile device,” indicating that more younger adults used ePHRs to download health information to computers or mobile devices (*P*=0.04).

**Table 2:** Description of the use of electronic personal health records among participants (N=1363).

Participant use of ePHR^a^ in the past 12 months	Total, n (%)	18-54 years old, n (%)	55 years old and above, n (%)	*P* values^b^
1. Request refill of medications	596 (46.57)	218 (44.0)	370 (51.8)	.13
2. Look up test results	1081 (84.59)	462 (84.2)	605 (85.3)	.75
3. Request correction of inaccurate information	104 (7.58)	39 (8.3)	63 (6.1)	.34
4. Securely message health care provider and staff (for example, email)	686 (52.96)	302 (54.9)	377 (49.0)	.10
5. Download your health information to your computer or mobile device, such as a cell phone or tablet	292 (25.88)	147 (28.7)	144 (20.1)	*.04* ^c^
6. Add health information to share with your health care provider, such as health concerns, symptoms, and side effects	307 (23.58)	134 (24.0)	173 (22.8)	.74
7. Help you make a decision about how to treat an illness or condition	324 (24.77)	130 (24.7)	188 (25.0)	.94
8. Electronically send health information	108 (9.62)	65 (10.6)	41 (7.7)	.26

^a^ePHR: electronic personal health record.

^b^*F* test was used for all items.

^c^Italics indicate *P*<.05.

### Weighted Multiple Linear Regression on Human-Technology Interaction Factors in Predicting ePHR Use Between 2 Age Groups

The regression analysis included 494 participants between 18 and 54 years old and 610 participants older than 55 years old. According to Table 3, at least 2 of the human-technology interaction factors of ePHRs were associated with the use of ePHRs among participants in both age groups. Including clinical notes was positively related to ePHR use in those 18 to 54 years old (*β*=.28; *P*=.005) and those 55 years old and older (*β*=.15*; P*=.006). Although accessing ePHRs using a smartphone app was only associated with ePHR use among younger adults (*β*=.29; *P*<.001), ease of understanding health information in ePHRs was positively linked to ePHR use only among older adults (*β*=.13; *P*=.003)*.* Other than some human-technology interaction factors, having a regular health care provider and having friends or family to talk to about health were positively associated with the use of ePHRs among younger adults.

**Table 3:** Weighted multiple linear regression on human-technology interaction factors predicting electronic personal health record use between 2 age groups.

Predictor of use of ePHRs^a^			Standardized coefficient for 18-54 years old (*β*)		*P* values	Standardized coefficient for 55 years old and older (*β*)				*P* values
**Sociodemographics**										
	Gender: female (ref^b^=male)	.01		.89			–.06		.27	
	Urbanity: urban (ref=rural)	.08		.40			.04		.76	
	Education: bachelor’s degree and above (ref=below bachelor’s degree)	–.05		.44			.11		.44	
**Health care resources**										
	Regular health care provider: yes (ref=no)	.14		.04			.12		.08	
	Frequency of visiting health care provider: 4 times and above (ref=0-3 times)	.02		.31			.05		.36	
	Having friends/family to talk to about health: yes (ref=no)	.12		.005			.10		.19	
**Human-technology interaction factors**										
	Accessing ePHRs using smartphone app: yes (ref=no/don’t know)	.29		<.001			.10		.14	
	ePHRs include clinical notes: yes (ref=no/don’t know)	.28		.005			.15		.006	
	Ease of understanding ePHR health information (range:0-3)	–.01		.89			.13		.003	

^a^ePHRs: electronic personal health records.

^b^ref: reference.

## Discussion

### Principal Results and Comparison to Prior Work

Analyzing the most recent iteration of the HINTS collected in 2019, this study aimed to examine the relationship between human-technology interaction factors and ePHR use among adults and then to compare its different effects between younger adults (18-54 years old) and older adults (55 years old and older) while controlling for sociodemographics and health care resources.

### The Level of ePHR Use Among Younger Adults and Older Adults

This study found that the average level of ePHR use was low (mean 2.76, range 0-8). This is in line with Hong and colleagues’ [10] study that reported the use of ePHRs in the United States to be 31.4%. However, while Hong et al measured ePHR use by asking participants whether they had accessed ePHRs in the past 12 months (yes or no), our study only included participants who had accessed their ePHRs at least once in the past 12 months and measured the use level of different ePHR functions (eg, request refills of medications, look up test results, message health care provider and staff). This suggested that even among participants who accessed ePHRs, the use of ePHR functions is still low. The study also found that there was no significant difference in average ePHR use between the 2 age groups, which contradicts the findings of Greenberg et al [11] and McInnes et al [12], who reported younger age to be related to higher ePHR use. Including performance expectancy, effort expectancy, social influence, and facilitating conditions as independent variables, 2 studies conducted by Abd-Alrazaq and colleagues [29,30] found that age moderated the effects of performance expectancy, effort expectancy, and facilitating conditions on intention to use ePHRs. The moderating effect of age might be able to explain the nonsignificant finding of age difference in our study, which suggests that future studies are needed to explore the moderating effect of age using the current data set and measurements.

### Human-Technology Interaction Factors Associated With ePHR Use

In terms of human-technology interaction factors associated with ePHR use, this study found that including clinical notes was positively related to ePHR use in both age groups. Previous studies examining the relationship between including clinical notes and ePHR use rates were not found. Nonetheless, in a survey evaluating veterans’ access to an ePHR program called My HealtheVet Pilot, participants reported the highest rates (585/657, 89%) on using patient records including clinical notes or lab test results, and participants perceived that viewing medical records including clinical notes was the most useful feature of the ePHR programs [31]. In a qualitative study exploring participants’ views on the My Health*e*Vet Pilot, participants identified that clinical notes promoted active patient participation by helping them prepare for the clinical visit, gain insight about their health and treatment plans, and gain insight into the providers’ perspectives [32].

Regarding the different effects of human-technology interaction factors on ePHR use between the 2 age groups, accessing ePHRs using a smartphone app was significantly associated with ePHR use among younger adults while ease of understanding health information in ePHRs was significantly linked to ePHR use among older adults.

In terms of accessing ePHRs using a smartphone app, our findings are consistent with Bell et al’s [33] findings that indicate accessing ePHRs through a mobile app to be associated with higher ePHR use. However, a conflicting finding was found in 2 previous studies: using the ePHRs only via a mobile device was related to infrequent use of ePHRs [34,35]. This discrepancy might be the result of samples with different characteristics in different studies being used. The study from Bell et al [33] was conducted among adults after elective orthopedic surgery, the study from Graetz et al [34] was conducted among adult patients with diabetes, and the study from Jung et al [35] was conducted with adults in South Korea. Moreover, previous studies showed that younger participants are more likely to use ePHRs only via a mobile device [33,34], which was also found in our study. The bivariate analysis of our study also indicated that younger adults were more likely to download health information to computers or mobile devices, such as a cell phone or tablet, which highlighted the significant role of mobile devices in ePHR use among younger adults.

Another human-technology interaction factor, ease of understanding health information in ePHRs, was found to be significantly linked to ePHR use among older adults but not younger adults. This finding is in line with Abd-Alrazaq et al’s [29,30] studies, which suggested that perceived ease of use is positively associated with the intention to use ePHRs, with this relationship being stronger among older patients. This difference might be explained by the lower health literacy among older adults compared to their younger counterparts [36].

### Conclusions

The purpose of this cross-sectional study was to examine the relationship between human-technology interaction factors and ePHR use among adults and then compare its different effects between younger adults (18-54 years old) and older adults (55 years old and older). The study found that the average level of ePHR use was low and that there was no significant difference in average use of ePHRs between the 2 age groups. Regarding the human-technology interaction factors, including clinical notes was positively related to ePHR use in both age groups, and accessing ePHRs using a smartphone app was positively associated with ePHR use among younger adults, while ease of understanding health information in ePHRs was a positive factor for ePHR use among older adults. The current study showed that there is a significant relationship between human-technology interaction factors and ePHR use and that the human-technology interaction factors associated with ePHR use vary across different age groups. In order to broadly promote the use of ePHRs, the design of ePHRs should take significant human-technology interaction factors into consideration, and the education or training regarding ePHR use should be provided for both health care providers and patients, especially for older adults.

### Limitations

There are several limitations of this study. First, this study used a cross-sectional data set that was not able to examine causality between human-technology interaction factors and ePHR use. Second, the this study only included participants who were offered accesses to their ePHRs and accessed their ePHRs at least once in the past 12 months. Only 34% of US adults reported that they were offered access to their ePHRs [37], and, of those patients who were offered ePHR access, only 30% of patients actually accessed their ePHRs at least once in a year [11]. In order to promote meaningful use of ePHRs and maximize the benefit of ePHRs for patients, future studies may explore the factors that affect offering ePHR access to patients and patients’ not accessing ePHRs even with access being granted. Finally, ePHR use in this study was measured by 8 self-reported items regarding the purposes for which participants used ePHRs, which might not have accurately recorded the actual use of ePHRs among participants. Future studies may consider using data including the frequencies and times that participants login to their ePHR accounts.

### Implications for Practice and Future Research

Despite these limitations, this study is the first of its kind to examine the association between human-technology interaction factors and ePHR use among US adults and to compare its different effects between younger adults (18-54 years old) and older adults (55 years old and older). The findings of this study provide implications for practice and future research. This study found that including clinical notes was positively related to ePHR use in both age groups, which suggests that including clinical notes as a part of ePHRs might improve the effective use of ePHRs among patients. Although clinical notes can serve as a fundamental feature for ePHRs, participants in Woods et al’s [32] study also demonstrated difficulties in seeing clinical notes, such as the use of derogatory terms, stress when seeing detailed personal information, and challenging conversations with providers. Although our study only examined “including clinical notes” as a single item, future studies are needed to explore patients’ preferences on the type of clinical notes that should be included in ePHRs. This will also maximize the meaningful use of clinical notes. Moreover, this study found that accessing ePHRs using a smartphone app was associated with higher ePHR use among younger adults while ease of understanding health information in ePHRs was linked to higher ePHR use among older adults. The design of ePHRs should provide the option of being accessible through mobile devices to promote greater ePHR use among young people. For older adults, providers could add additional notes to explain the health information recorded in the ePHRs.

Empirical evidence has demonstrated that ePHRs provide consumers with easy and convenient access to their health data [1]. As the landscape of personal health care delivery changes due to increased technological advancements, there will be continued use of ePHRs. By addressing the concerns related to clarity in clinical notes for older adults and a simpler app platform for younger adults, ePHRs can increase access to health care data for both younger and older adults. With this increased use and access, it is important to highlight the benefits of using ePHRs in rural communities. Rural communities are often racially diverse, older, and tend to have lower incomes with limited access to health care [38]. In rural communities, telehealth is being used to address inequities in health care. Coupled with telehealth options, ePHRs can provide greater access to health data for individuals who reside in rural communities. This ease of access is also critical during times of prolonged crises, such as a pandemic. Since the coronavirus outbreak in March 2020 in the United States, the country has dealt with unprecedented circumstances in the medical field as medical staff continue to serve patient's routine and emergent health care needs. The use of and access to health care data through ePHRs has allowed patients to stay in touch with their providers while allowing protective social distancing measures to remain in place, especially for older adults who are at higher risk. Properly educating physicians and consumers on the benefits of ePHRs and how to use ePHRs to access data at any time will increase communication between the physicians and consumers. This will also aid consumers in adjusting to changes in health care delivery as it allows them to continue to feel connected to their health care provider during such a critical time in health care. During a pandemic, voluntary participation in data sharing via ePHRs would allow health authorities access to critical data on medical diagnoses that indicate who is at an elevated risk for additional negative impacts from COVID-19 [39]. This access could allow for valuable protective measures to be extended for at-risk populations and keep health authorities apprised of the success or failure of proactive measures to protect these higher-risk groups.

## Abbreviations

ePHR: electronic personal health record
HINTS: Health Information National Trends Survey
PHR: personal health record
